# CellWalker: a user-friendly and modular computational pipeline for morphological analysis of microscopy images

**DOI:** 10.1093/bioinformatics/btad710

**Published:** 2023-12-07

**Authors:** Harshavardhan Khare, Nathaly Dongo Mendoza, Chiara Zurzolo

**Affiliations:** Membrane Traffic and Pathogenesis Unit, Department of Cell Biology and Infection, CNRS UMR 3691, Université de Paris, Institut Pasteur, Paris, 75015, France; Membrane Traffic and Pathogenesis Unit, Department of Cell Biology and Infection, CNRS UMR 3691, Université de Paris, Institut Pasteur, Paris, 75015, France; Centro de Investigación en Bioingeniería – BIO, Universidad de Ingeniería y Tecnología – UTEC, Lima, 15063, Perú; Membrane Traffic and Pathogenesis Unit, Department of Cell Biology and Infection, CNRS UMR 3691, Université de Paris, Institut Pasteur, Paris, 75015, France; Department of Molecular Medicine and Medical Biotechnology, University of Naples Federico II, Naples, 80131, Italy

## Abstract

**Summary:**

The implementation of computational tools for analysis of microscopy images has been one of the most important technological innovations in biology, providing researchers unmatched capabilities to comprehend cell shape and connectivity. While numerous tools exist for image annotation and segmentation, there is a noticeable gap when it comes to morphometric analysis of microscopy images. Most existing tools often measure features solely on 2D serial images, which can be difficult to extrapolate to 3D. For this reason, we introduce CellWalker, a computational toolbox that runs inside Blender, an open-source computer graphics software. This add-on improves the morphological analysis by seamlessly integrating analysis tools into the Blender workflow, providing visual feedback through a powerful 3D visualization, and leveraging the resources of Blender's community. CellWalker provides several morphometric analysis tools that can be used to calculate distances, volume, surface areas and to determine cross-sectional properties. It also includes tools to build skeletons, calculate distributions of subcellular organelles. In addition, this python-based tool contains ‘visible-source’ IPython notebooks accessories for segmentation of 2D/3D microscopy images using deep learning and visualization of the segmented images that are required as input to CellWalker. Overall, CellWalker provides practical tools for segmentation and morphological analysis of microscopy images in the form of an open-source and modular pipeline which allows a complete access to fine-tuning of algorithms through visible-source code while still retaining a result-oriented interface.

**Availability and implementation:**

CellWalker source code is available on GitHub (https://github.com/utraf-pasteur-institute/Cellwalker-blender and https://github.com/utraf-pasteur-institute/Cellwalker-notebooks) under a GPL-3 license.

## 1 Introduction

One of the most significant technological advancements in biology is the use of computer tools for the processing of microscopy images, providing unprecedented opportunities to understand cell morphology and connectivity, and investigate structure-function relationships. For example, the reconstruction of microscopy images of tissues has facilitated quantification of neuronal structures such as synapses and dendrites, subcellular organization of organelles like mitochondria, endoplasmic reticulum, and microtubules (Tamada *et al.* 2017, Jorstad *et al.* 2018, Kubota *et al.* 2018, Polo *et al.* 2020, Feng *et al.* 2023). Throughout the past few years, image analysis programs have benefited the study of life sciences.

Currently, a wide range of open-source tools are available for image processing, classification, and segmentation. Notable examples include MIB (Belevich *et al.* 2016), KNOSSOS (Helmstaedter *et al.* 2011), AxonSeg (Zaimi *et al.* 2016), SegEM (Berning *et al.* 2015), CATMAID (Saalfeld *et al.* 2009), gACSON (Behanova *et al.* 2022), and VAST (Berger *et al.* 2018). These software tools offer a comprehensive set of functionalities, including image transformation, filtering, and normalization which are described in Supplementary Table S1. Some of these tools have been updated to incorporate advanced segmentation features such as DeepMIB (Belevich and Jokitalo 2021), Webknossos (Zaimi *et al.* 2018), and AxonDeepSeg (Zaimi *et al.* 2018).

However, despite the abundance of image annotation and segmentation tools, there is a noticeable gap in the field of morphometric analysis for microscopy images, particularly for access to 3D visualization and analysis of biological objects. In addition, it is worth mentioning that specific tools like AxonSeg (Zaimi *et al.* 2016), AxonDeepSeg (Zaimi *et al.* 2018), SegEM (Berning *et al.* 2015), Neuromorph (Jorstad *et al.* 2018), and gACSON (Behanova *et al.* 2022), are primarily designed for neuronal circuit microscopy analysis. Existing tools like Webknossos and MIB often limit feature measurements to 2D serial images (Belevich *et al.* 2016, Boergens *et al.* 2017). A tool specifically designed for morphometry of objects in segmented microscopy images can be effective to address the need for generalized and simplified morphological analyses.

Therefore, we introduce the CellWalker package that leverages the 3D space of Blender. Its unique features allow users to perform 3D operations and visualize cellular/subcellular objects in segmented microscopy images. Moreover, the CellWalker can be integrated with other Blender add-ons and tools, enabling the exploration and utilization of a wide range of morphological analysis techniques. We utilize existing open-source technologies to make CellWalker visible-source wherever possible. This approach allows users to use customized input data, tweak the algorithms and employ the in-built tools in the software packages inside which the CellWalker modules run.

## 2 Materials and methods

**Figure 1.**
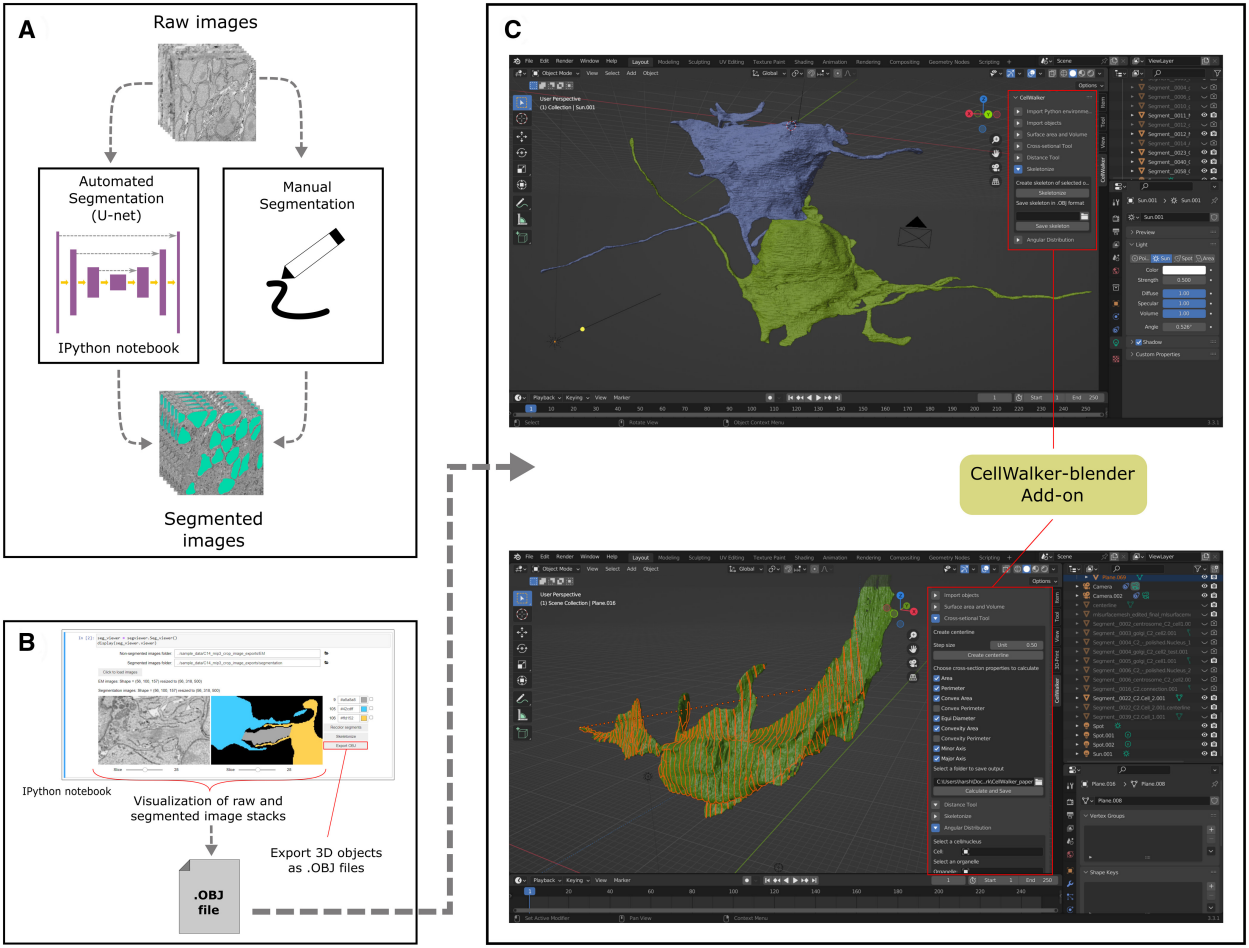
(A, B) Preparation of input for C (CellWalker); (A) segmentation using provided IPython notebook for automated segmentation using UNET (left) or manual segmentation (right); (B) visualization of segmentation and exporting 3D objects as.OBJ files using provided IPython notebook; (C) CellWalker (Blender add-on) shown inside Blender interface—two example cells loaded as 3D objects (top) and cross-sectioning performed on an example cell (bottom).

CellWalker is written in python (version ≥ 3.10). It consists of helper IPython notebooks (Fig. 1A and B) for segmentation utilities and the main morphology analyzer tool that runs as an add-on inside the Blender 3D graphics software (Fig. 1C). The pipeline can be divided into three modules—Image Segmentation, Visualization and Exportation and Morphological Analysis. There are several steps involved in each of these stages (please see Supplementary Information and the GitHub repositories).

### 2.1 Morphological analysis

CellWalker is provided as an add-on of the Blender software (Fig. 1C). Blender is an open-source 3D graphics software that allows rendering and processing of 3D objects. CellWalker takes advantage of existing Blender user interface and 3D modeling capabilities to provide a toolbox of functions more suited to cell biological applications while retaining the possibility of using existing functionalities of Blender. CellWalker provides several tools for morphological characterization of biological 3D objects. At the same time, the user interface of Blender offers fluent 3D rendering.

CellWalker can be installed as a standard Blender add-on [please refer the user-guide (https://github.com/utraf-pasteur-institute/CellWalker-blender/wiki for more details)]. Once installed, the CellWalker add-on can be found in the Sidebar of the Blender’s interface which can be opened by pressing ‘N’ on the keyboard.

CellWalker provides several tools for morphological analysis as described below.

#### 2.1.1 Importing objects

This tool helps in importing 3D objects from .OBJ files. It allows bulk import of all .OBJ files inside a folder to reduce manual efforts. The import functionality also tries to assign correct orientation to the objects while importing.

#### 2.1.2 Surface and volume

Surface area and volume calculations are facilitated by this function. The function calculates surface area and volume of one or more selected objects using the mesh obtained from the .OBJ file.

#### 2.1.3 Cross-sectioning and cross-section properties

Biological objects commonly have nonuniform shapes. Understanding these shapes is improved by visualizing the cross-sections in a specific orientation. The cross-sectioning tool provides an easy way to define a center line along which cross-sections are created. In addition, this tool also allows calculation of geometric properties of the cross-sections including area, perimeter, convex area, convex perimeter, equivalent diameter, convexity in terms of area, convexity in terms of perimeter, minor axis length, and major axis length.

#### 2.1.4 Skeletonization

This unique functionality offers building skeletons of 3D biological objects. Skeletons are topology preserving thin line representations of biological structures. Such representations can give deeper insights into the shapes of objects and might help in understanding the correlations with their functions. The CellWalker provides an implementation of the Kimimaro skeletonization method (William Silversmith *et al.* 2021), into Blender’s 3D rendering making the algorithm easily accessible during visualization of cellular structures.

#### 2.1.5 Distance calculation

Distance calculation is one of the most fundamental aspects of inference in structural cellular biology. CellWalker can calculate distance between any two selected points on the mesh surface of loaded objects. CellWalker measures two types of distances—straight distance and surface distance. The straight distance is conventional straight-line distance through space. The surface distance is the path length required to reach the other point by traversing on the surface of the object. This surface distance is calculated using Dijkstra’s shortest path calculation on the object’s mesh representation.

#### 2.1.6 Angular distribution of objects

Angle distribution of objects describes the spread of the objects of interest around a chosen point. For example, distribution of Golgi apparatus around the center of the nucleus can be an important factor for inferences about the stages in the cell cycle. Another example is the distribution of vesicles with reference to the center of the cell body, which can be important in neuronal synaptic communication. The calculation of angular distribution requires an object of interest and a reference point around which distribution is to be calculated. The calculated values are the mean value of angles subtended at the reference point with respect to a fixed point in the object of interest and the standard deviation of those angles. Larger mean value of the angles indicates broader spread of the object around the reference point, whereas the larger standard deviation suggests more uniform spread of the object of interest.

### 2.2 Creating input images for CellWalker

To assist creation of segmented images required as input to the CellWalker Blender add-on, we provide image segmentation and segmentation visualization scripts as IPython Notebooks (https://github.com/utraf-pasteur-institute/CellWalker-notebooks) that are open-source as well as visible-source, enhancing the flexibility and accessibility for computational biologists while keeping the code execution sufficiently simple for noncoding users. The IPython notebooks can also be freely executed on cloud computing platforms like Google Colaboratory.

#### 2.2.1 Image segmentation

The automated segmentation notebook implements a 2D UNET algorithm (Ronneberger *et al.* 2015). After training on manually segmented ground truth images, this 2D-UNET is applied to every slice of a 3D image to obtain a 3D segmentation (Fig. 1A). The segmentation output is generated as a stack of 2D segmented images (Fig. 1A) which can then be supplied to the segmentation viewer notebook for visualization.

#### 2.2.2 Visualization and exporting

The segmentation visualizer (Fig. 1B) can display original and segmented images side-by-side for direct confirmation of the quality of segmentation. It is possible to set colors of the segmented objects to assist in visualization of multi-object segmentations. The segmentation visualizer also provides a function to transform voxelated 3D segments into 3D meshes using a marching cubes algorithm (Lorensen and Cline 1987). These meshes are exported as 3D models in a generic Wavefront OBJ format (Fig. 1B) which is compatible with other modeling and simulation programs including Blender and Autodesk 3ds Max. Biologists frequently resort to manual segmentation due to lack of sufficient training data for deep learning methods, high cost of generating ground-truth data (Sun *et al.* 2022), and need for re-training the algorithms for different morphologies, resolution, image quality, or acquisition techniques (Meijering 2020, Pelt 2020). The segmentation viewer can be used to export 3D objects from such manually segmented images as well. The objects exported as the .OBJ files can be then analyzed in the CellWalker Blender add-on (Fig. 1C).

## 3 Case study

We provide a case study to demonstrate the utility of the CellWalker in calculation of morphological features which may be useful for comparative analysis of interconnected Granule Cells in developing mouse cerebellum at various stages as described by Cordero-Cervantes *et al.* (2023). The calculations have been performed on serial sectioning scanning electron microscopy data obtained from mouse cerebellum at postnatal days 0: https://zenodo.org/records/7594891#.Y9qnNHbMKUk (Cordero-Cervantes *et al.* 2023), 3 and 7: https://bossdb.org/project/wilson2019 (Wilson *et al.* 2019). This quantitative comparison of examples from all three developmental stages (P0, P3 and P7) was not available in the report by Cordero-Cervantes *et al.* (2023). We describe various biologically relevant measurements such as dimensions of lamellipodial and filopodial protrusions (including length, major axis, minor axis, convexity, cross-sectional area), volumes of cells at different developmental stages, diameters, and lengths of interconnections for different cell pairs, and distribution of Golgi apparatus at various postnatal stages. The detailed results of the case study can be found at https://github.com/utraf-pasteur-institute/CellWalker-blender/wiki/Study-Case.

## 4 Conclusion

We designed a python-based toolbox, named CellWalker, for morphological analysis of microscopic 3D images of cells. While currently developed for cell-biological applications, the CellWalker may also be extended to tissue or organ-level analyses. Our toolbox bridges the segmentation protocols with 3D morphological measurements while keeping the corresponding modules independently accessible. Optionally, we provide ‘visible-source’ IPython notebooks accessories for segmentation of 2D/3D microscopy and object reconstruction. This modular approach allows us to take advantage of existing computing infrastructures and tools such as Google Colab, IPython notebooks and the Blender software package. The design of CellWalker is also kept open-source and visible-source as much as possible to make it useful for both programmers and noncoding users.

## Supplementary Material

btad710_Supplementary_Data

## Data Availability

CellWalker source code is available on GitHub (https://github.com/utraf-pasteur-institute/Cellwalker-blender and https://github.com/utraf-pasteur-institute/Cellwalker-notebooks) under a GPL-3 license. All data supporting this article is also available in these repositories.
