# Supplementary material for: CellWalker: a user-friendly and modular computational pipeline for morphological analysis of microscopy images
Source: Bioinformatics. 2023 Dec 7;39(12):btad710. doi: 10.1093/bioinformatics/btad710 (PMC10713108; doi:10.1093/bioinformatics/btad710)
Supplement: btad710_Supplementary_Data [file btad710_supplementary_data.zip › Supplementary_Table.docx]

**Table 1. Open-source software tools for microscopy image analysis**

| **Neuromorph** (Jorstad et al 2018) | Neuromorph works inside Blender as an add-on and leverages Blender’s 3D graphics environment (Jorstad et al., 2018). It is specifically designed for analysis of neuronal microscopic images with various functionalities including length measurements, surface area, volume calculation, cross-sectioning, proximity analysis and 3D drawing. It also provides tools to create segmentations inside Blender, however users have to mainly depend on external tools such as ImageJ for creating segmented images needed as input to Neuromorph. |
| --- | --- |
| **Microscopy Image Browser**  (Belevich et al., 2016) | Microscopy Image Browser (MIB) is a software package designed for efficient segmentation and image processing of multidimensional datasets. It provides a comprehensive set of tools for image transformation, filtering, and normalization, supporting various image formats. Furthermore, MIB includes DeepMIB (Belevich & Jokitalo, 2021), an additional feature accessible from the menu, which allows users to train convolutional neural networks for segmentation tasks. |
| **KNOSSOS** (Helmstaedter et al., 2011) | KNOSSOS, initially developed as a browsing and annotation tool for large-scale electron microscopy (EM) multidimensional data, utilizes skeletons and a redundant-skeleton consensus procedure algorithm. This enables users to navigate and annotate EM data effectively. Building upon KNOSSOS, the Webknossos tool (Boergens et al., 2017), enhances 3D EM annotation capabilities and incorporates a flight mode feature, which significantly reduces annotation time. |
| **AxonSeg**  (Zaimi et al., 2016) | AxonSeg is a MATLAB software that offers a comprehensive set of tools for axon annotation, segmentation, and morphological analysis. It specializes in automatic axon and myelin segmentation using a graph-based approach on histology images. Additionally, AxonSeg can measure various neuron morphometric parameters. In contrast, AxonDeepSeg (Zaimi et al., 2018), provides a more automated and reliable solution compared to AxonSeg. It eliminates the need for manual intervention in most cases by utilizing deep learning techniques for axon segmentation. |
| **SegEM**  (Berning et al., 2015) | SegEM is a comprehensive set of tools designed to analyze large-scale 3D electron microscopy (EM) datasets efficiently. It focuses on reconstructing neuronal circuits by integrating skeleton reconstructions of neurons with automated volume segmentations. SegEM also offers a reliable classifier selection process to identify the most suitable automated image classifier for different types of nerve tissue. |
| **Collaborative Annotation Toolkit for Massive Amounts of Image Data** (Saalfeld et al., 2009) | Collaborative Annotation Toolkit for Massive Amounts of Image Data (CATMAID) is a specialized software platform created for efficiently handling and analyzing large collections of biological image data. It offers a user-friendly interface like Google Maps, allowing users to easily navigate, collaborate, and annotate massive datasets. CATMAID supports 3D biological image data obtained through various microscopy techniques, such as optical or physical sectioning. It facilitates the sharing of specific areas of interest using bookmarks and enables synchronized navigation across multiple registered datasets. |
| **Volume Annotation and Segmentation Tool**  (Berger et al., 2018) | VAST, a C++ program, is a powerful tool for generating and editing annotations and segmentations of large volumetric image data sets. It supports grayscale and RGB image stacks, which can be imported into VAST's 3D data format or accessed locally as image tiles. It also allows streaming of image data from online sources. With the ability to load and display multiple image and segmentation layers, VAST is an excellent choice for voxel-based ground truth annotation through voxel painting. |

**References**

Belevich, I., Joensuu, M., Kumar, D., Vihinen, H., & Jokitalo, E. (2016). Microscopy Image Browser: A Platform for Segmentation and Analysis of Multidimensional Datasets. *PLoS Biology*, *14*(1). https://doi.org/10.1371/journal.pbio.1002340

Belevich, I., & Jokitalo, E. (2021). DeepMIB: User-friendly and open-source software for training of deep learning network for biological image segmentation. *PLoS Computational Biology*, *17*(3). https://doi.org/10.1371/journal.pcbi.1008374

Berger, D. R., Seung, H. S., & Lichtman, J. W. (2018). VAST (Volume Annotation and Segmentation Tool): Efficient manual and semi-automatic labeling of large 3D image stacks. *Frontiers in Neural Circuits*, *12*. https://doi.org/10.3389/fncir.2018.00088

Berning, M., Boergens, K. M., & Helmstaedter, M. (2015). SegEM: Efficient Image Analysis for High-Resolution Connectomics. *Neuron*, *87*(6). https://doi.org/10.1016/j.neuron.2015.09.003

Boergens, K. M., Berning, M., Bocklisch, T., Bräunlein, D., Drawitsch, F., Frohnhofen, J., Herold, T., Otto, P., Rzepka, N., Werkmeister, T., Werner, D., Wiese, G., Wissler, H., & Helmstaedter, M. (2017). WebKnossos: Efficient online 3D data annotation for connectomics. *Nature Methods*, *14*(7). https://doi.org/10.1038/nmeth.4331

Helmstaedter, M., Briggman, K. L., & Denk, W. (2011). High-accuracy neurite reconstruction for high-throughput neuroanatomy. *Nature Neuroscience*, *14*(8). https://doi.org/10.1038/nn.2868

Jorstad, A., Blanc, J., & Knott, G. (2018). NeuroMorph: A Software Toolset for 3D Analysis of Neurite Morphology and Connectivity. *Frontiers in Neuroanatomy*, *12*. https://doi.org/10.3389/fnana.2018.00059

Saalfeld, S., Cardona, A., Hartenstein, V., & Tomančák, P. (2009). CATMAID: Collaborative annotation toolkit for massive amounts of image data. *Bioinformatics*, *25*(15). https://doi.org/10.1093/bioinformatics/btp266

Zaimi, A., Duval, T., Gasecka, A., Côté, D., Stikov, N., & Cohen-Adad, J. (2016). AxonSeg: Open source software for axon and myelin segmentation and morphometric analysis. *Frontiers in Neuroinformatics*, *10*(AUG). https://doi.org/10.3389/fninf.2016.00037

Zaimi, A., Wabartha, M., Herman, V., Antonsanti, P. L., Perone, C. S., & Cohen-Adad, J. (2018). AxonDeepSeg: Automatic axon and myelin segmentation from microscopy data using convolutional neural networks. *Scientific Reports*, *8*(1). https://doi.org/10.1038/s41598-018-22181-4
